# Aging Does Not Exacerbate Muscle Loss During Denervation and Lends Unique Muscle-Specific Atrophy Resistance With Akt Activation

**DOI:** 10.3389/fphys.2021.779547

**Published:** 2021-11-30

**Authors:** Jae-Sung You, Jie Chen

**Affiliations:** Department of Cell and Developmental Biology, University of Illinois at Urbana-Champaign, Urbana, IL, United States

**Keywords:** aging, muscle, denervation, protein synthesis, rRNA, Akt, mTORC1

## Abstract

Sarcopenia, or age-related skeletal muscle atrophy and weakness, imposes significant clinical and economic burdens on affected patients and societies. Neurological degeneration, such as motoneuron death, has been recognized as a key contributor to sarcopenia. However, little is known about how aged/sarcopenic muscle adapts to this denervation stress. Here, we show that mice at 27months of age exhibit clear signs of sarcopenia but no accelerated denervation-induced muscle atrophy when compared to 8-month-old mice. Surprisingly, aging lends unique atrophy resistance to tibialis anteria muscle, accompanied by an increase in the cascade of mammalian target of rapamycin complex 1 (mTORC1)-independent anabolic events involving Akt signaling, rRNA biogenesis, and protein synthesis during denervation. These results expand our understanding of age-dependent stress responses and may help develop better countermeasures to sarcopenia.

## Introduction

Loss of skeletal muscle mass, or atrophy, can occur in a variety of conditions throughout life, including disuse, malnutrition, injury, diseases, and aging (You et al., 2015, 2018, 2021b). Age-related muscle atrophy and weakness (i.e., sarcopenia) in particular has rapidly become prevalent with increasing lifespan, and it is highly associated with loss of independence, an increased risk of morbidity and mortality, and a significant economic burden (Janssen et al., 2004; Larsson et al., 2019). In addition to its basal phenotypes, aged muscle exhibits distinct physiological and molecular responses to various stresses, such as overload, immobilization, and retraining/reloading after disuse (Hwee and Bodine, 2009; Suetta et al., 2009; Miller et al., 2019). Expanding our understanding of such age-dependent adaptations will help develop better countermeasures to sarcopenia.

The etiology of sarcopenia is complex and multifactorial, involving both cell-intrinsic and -extrinsic (i.e., non-muscle cell) changes that are closely intertwined (Larsson et al., 2019; Soendenbroe et al., 2021). Among the extrinsic factors, progressive perturbations in the motoneuron system (from pre-synaptic degeneration to motoneuron death or denervation) have been recognized as the key contributor to the disease state (Hepple and Rice, 2016; Larsson et al., 2019). In young/adult mice, denervation *via* surgical dissection of the sciatic nerve reduces muscle mass up to 50% within 2weeks (Tang et al., 2014; You et al., 2021a). This drastic rate of atrophy is comparable to the accelerated atrophy of human muscle and physical frailty that begin with increasing frequency of denervation in ages over 75years (Hepple and Rice, 2016). While it seems clear that spontaneous denervation accelerates sarcopenia in advanced age, little is known about whether aged muscle *per se* is more susceptible to the loss of neural input than younger muscle. If more susceptible, such intrinsic vulnerability would be an additional factor contributing to sarcopenia.

In general, skeletal muscle mass is determined by a net balance between the global rates of protein synthesis and protein degradation (Goodman et al., 2011b). Muscle atrophy that occurs in response to acute denervation is also regulated by the changes in the rates of protein synthesis and protein degradation (Quy et al., 2013; You et al., 2018, 2021a). It is well known that signaling molecules governing anabolism and/or catabolism such as the mammalian target of rapamycin complex 1 (mTORC1) and Akt play a critical role in modulating the severity of denervation-induced atrophy (Bodine et al., 2001; Quy et al., 2013; You et al., 2021a). Yet, how these regulators and protein metabolism change upon denervation in aged muscle is not understood.

The current study aimed to determine the effects of aging on the skeletal muscle responses to denervation. Our results show that aging does not promote muscle susceptibility to denervation-induced atrophy and surprisingly lends atrophy resistance to a specific type of muscle through enhanced anabolic signaling.

## Materials and Methods

### Animals

All animal experiments were performed in compliance with protocols approved by the Institutional Animal Care and Use Committee at the University of Illinois at Urbana-Champaign (#19255). Wild-type C57BL/6N male mice were maintained in an EcoFlo ventilation cage system (Allentown Inc., Allentown, NJ, United States) at 23°C with a 12-h light/dark cycle and fed *ad libitum*. Mice at 8 and 27months of age received denervation surgery as described below, and any mice exhibiting signs of abnormality during the recovery period, such as lethargy and reduced body weight, were excluded from the study. The animals were anesthetized with isoflurane during all surgical and euthanasia procedures.

### Denervation

Unilateral denervation of the hindlimb muscles was performed by excising an approximately 0.5-cm-long segment of the sciatic nerve from the right leg. The left contralateral control leg was subjected to a sham surgery that involved only an incision in the skin where the denervation surgery would have occurred. After the surgery, the skin incision was closed with a 3-0 polysorb suture.

### Measurements of Protein Synthesis

*In vivo* measurements of protein synthesis rates were performed with the surface sensing of translation (SUnSET) technique (Goodman et al., 2011a). Specifically, puromycin (75mM stock in diH_2_O) was diluted in 200μl of PBS and intraperitoneally injected into mice at the concentration of 0.04μmol/g bodyweight. Muscles were collected exactly 30min after injection and analyzed by Western blotting for the amount of puromycin-labeled peptides per mg protein or per muscle.

### Sample Preparation and Protein Content

Collected muscles were immediately cut in half cross-wise and each was weighed and frozen in liquid nitrogen for storage. The proximal halves of the muscles were homogenized with a Polytron in ice-cold buffer containing 20mM Tris (pH 7.4), 0.3% Triton X-100, 2mM EGTA, 2mM EDTA, 0.1mM Na_3_VO_4_, 25mM NaF, 25mM β-glycerolphosphate, and 1×protease inhibitor cocktail (MilliporeSigma, P8240; Burlington, MA, United States). Protein concentrations of the homogenates were measured with the DC protein assay kit (Bio-Rad, Hercules, CA, United States), and total protein content in each whole muscle was calculated.

### Western Blotting

An equal amount of protein from each sample was boiled in Laemmli buffer, resolved by SDS-PAGE, transferred onto PVDF membrane, blocked with 5% milk in PBS-T (PBS with 0.5% Tween 20), and probed with appropriate primary and secondary antibodies listed below. After washing in PBS-T, blots were visualized and captured with the SuperSignal West Pico PLUS Chemiluminescent Substrate and an iBright CL1000 Imaging System (both from ThermoFisher Scientific, Waltham, MA, United States). The quantification of the blots was performed using ImageJ 1.53 (NIH, Bethesda, MD, United States; https://imagej.nih.gov/ij/).

### Antibodies

The antibodies used in this study are: anti-ubiquitin (Cat# sc-8017, RRID: *AB_2762364*), anti-IGF-1Rβ (Cat# sc-713, RRID: *AB_671792*), and anti-phospho-IRS-1 (Tyr632; Cat# sc-17196, RRID: *AB_669445*) from Santa Cruz Biotechnology (Dallas, TX, United States); anti-puromycin (Cat# MABE343, RRID: *AB_2566826*) from MilliporeSigma; anti-K48-linkage specific polyubiquitin (Cat# 8081, RRID: *AB_10859893*), anti-pan-actin (Cat# 8456, RRID: *AB_10998774*), anti-phospho-p70 S6 kinase (Thr389; Cat# 9234, RRID: *AB_2269803*), anti-p70 S6 kinase (Cat# 9202, RRID: *AB_331676*), anti-phospho-S6 (Ser240/244; Cat# 5364, RRID: *AB_10694233*), anti-S6 (Cat# 2317, RRID: *AB_2238583*), anti-phospho-ERK (Ser240/244; Cat# 4370, RRID: *AB_2315112*), anti-ERK (Cat# 9102, RRID: *AB_330744*), anti-phospho-Akt (Thr308; Cat# 13038, RRID: *AB_2629447*), anti-phospho-Akt (Ser473; Cat# 9271, RRID: *AB_329825*), anti-Akt (Cat# 9272, RRID: *AB_329827*), anti-phospho-glycogen synthase kinase-3β (GSK-3β; Ser9; Cat# 5558, RRID: *AB_10013750*), anti-GSK-3β (Cat# 9832, RRID: *AB_10839406*), anti-phospho-IGF-1Rβ (Tyr1135/1136; Cat# 3024, RRID: *AB_331253*), anti-IRS-1 (Cat# 2382, RRID: *AB_330333*), anti-phosphatidylinositol 3-kinase (PI3K) p85 (Cat# 4292, RRID: *AB_329869*), and anti-phosphatase and tensin homologue deleted on chromosome ten (PTEN; Cat# 9552, RRID: *AB_10694066*) from Cell Signaling Technology (Danvers, MA, United States); peroxidase-conjugated anti-rabbit IgG (Cat# 115-036-003, RRID: *AB_2617176*), anti-mouse IgG (Cat# 111-036-003, RRID: *AB_2337942*), and anti-mouse IgG2a (Cat# 115-035-206, RRID: *AB_2338514*) from Jackson Immuno Research Laboratories (West Grove, PA, United States); and peroxidase-conjugated anti-goat IgG (Cat# A27014, RRID: *AB_2536079*) from ThermoFisher Scientific.

### Total and Ribosomal RNA Content

The distal halves of the muscles were homogenized with a ribonuclease-free disposable pestle in 500μl of ice-cold TRIzol (Invitrogen), and 200μl of the upper aqueous phase was isolated for total RNA extraction. Total RNA was extracted using GeneJET RNA Purification Kit (ThermoFisher Scientific) with the first and second elution volume of 60 and 150μl, respectively, and the concentration of RNA eluates was measured by NanoDrop2000 (ThermoFisher Scientific). The gross amount of total RNA was normalized by the muscle weight. To measure ribosomal RNA (rRNA) content, RNA samples from equivalent amounts of muscle were run on 1.2% agarose gels, and the 28S and 18S rRNA were quantified by densitometry using ImageJ 1.53.

### qRT-PCR

Total RNA was reverse-transcribed into cDNA using qScript cDNA Synthesis Kit (Quanta Bioscience, Beverly, MA, United States). Gene expression was analyzed by qPCR on a StepOnePlus Real-Time PCR System (Applied Biosystems, Foster City, CA, United States) using SYBR green and primers listed in Table 1. *TATA-binding protein* (*Tbp*) was used as an internal control.

**Table 1:** qPCR primer list.

Gene		Sequence (5'→3')
*Chrna1*	Forward	GCACACACCATCACCATTTC
	Reverse	GGCTTCTTGGTCTGGGTAAA
*Chrnd*	Forward	CTTGTCTATGACTCGGGCTATG
	Reverse	CGAAAGGGAAGTAGGTGACTG
*Chrne*	Forward	GAGGAGGTGGAGTTCATCTTTG
	Reverse	CCCATTCTCCATTCTCGGTAAA
*Musk*	Forward	GTCTTCTTCAACACCTCCTACC
	Reverse	TCACAGCCTTCAGCTCATTC
*Hdac4*	Forward	GCACACACCATCACCATTTC
	Reverse	GGCTTCTTGGTCTGGGTAAA
*Myog*	Forward	CCACAATCTGCACTCCCTTAC
	Reverse	TCTCAGTTGGGCATGGTTTC
*Tbp*	Forward	GGGATTCAGGAAGACCACATAG
	Reverse	CCTCACCAACTGTACCATCAG

### Statistics

All values were presented as mean±SEM. A sample value that deviated more than three times from the mean in a given group was excluded as an outlier. Statistical significance (*p*<0.05) was determined by a two-tailed unpaired *t* test or two-way mixed ANOVA followed by a Tukey’s *post hoc* analysis. All statistical analyses were performed upon the verification of the test assumptions using the OriginPro 2021b software (OriginLab, Northampton, MA, United States).

## Results

### Effects of Aging on Denervation-Induced Muscle Atrophy

**Figure 1:**
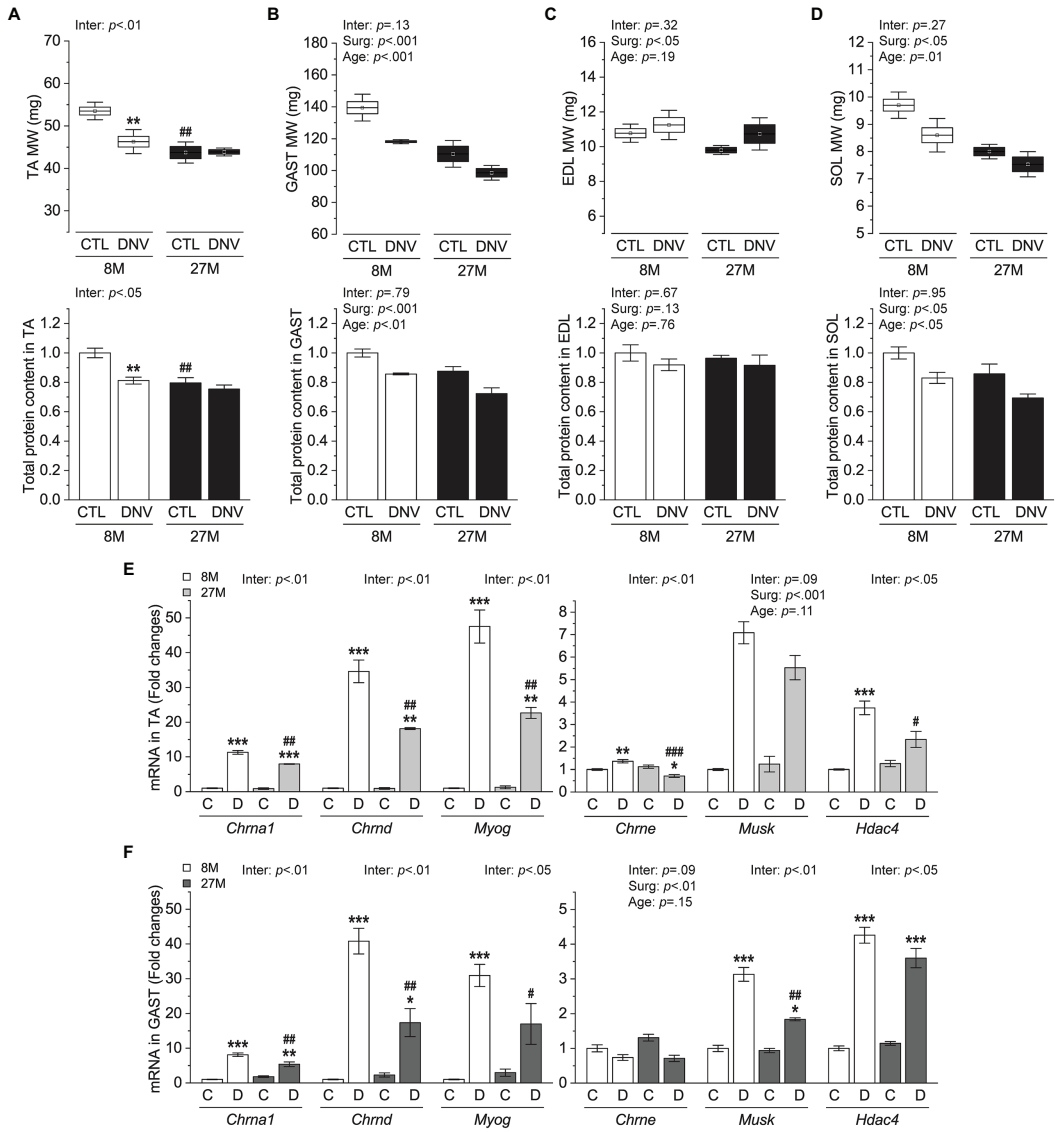
Effects of aging on denervation-induced muscle atrophy. Mice at 8months (8M) and 27months (27M) of age were subjected to denervation (right leg, DNV or D) and sham (contralateral left leg, CTL or C) surgeries, and tibialis anterior (TA), gastrocnemius (GAST), extensor digitorum longus (EDL), and soleus (SOL) muscles were collected 7days after surgeries. **(A–D)** Muscle weight and total protein content in TA **(A)**, GAST **(B)**, EDL **(C)**, and SOL **(D)**. **(E,F)** mRNA levels of denervation markers in TA **(E)** and GAST **(F)**. *n*=5–3 mice/group. Data are presented as mean±SEM. and SD (whisker, upper panels in **A–D**) and expressed relative to 8M/CTL except for upper panels in **(A–D)**. ^*^p<0.05, ^**^p<0.01, and ^***^p<0.001 vs. CTL by two-way mixed ANOVA. ^#^*p*<0.05, ^##^*p*<0.01, and ^###^*p*<0.001 vs. 8M within the same surgery groups by two-way mixed ANOVA. Inter, interaction between surgery effects (Surg) and age effects by two-way mixed ANOVA.

In this study, we compared 27-month-old mice (aged) to 8-month-old mice (adult) to investigate aging effects. The aged mice had a similar body weight as adult control mice (Supplementary Figure S1), but they showed apparent signs of sarcopenia as revealed by reduced muscle mass and total protein content in tibialis anteria (TA, Figure 1A), gastrocnemius (GAST, Figure 1B), extensor digitorum longus (EDL, Figure 1C), and soleus (SOL, Figure 1D) muscles. After 7days of denervation, adult mice lost a significant amount of muscle mass and total protein content in the TA; however, these denervation-induced atrophic responses did not occur prominently in aged mice (Figure 1A). In other muscles except for EDL, denervation reduced muscle mass and total protein content to a similar degree in adult and aged mice (Figures 1B–D). These results indicate that aged muscle is not more susceptible to denervation atrophy than younger muscle and rather exhibits atrophy resistance in a specific type of muscle.

We wondered whether the TA muscles of the 27-month-old mice had already undergone age-related denervation and became less sensitive to the surgical denervation than other muscles such as GAST. Hence, we measured several gene markers of denervation, including acetylcholine receptor (*Chrn*) subunits, muscle-specific kinase (*Musk*), Myogenin (*Myog*), and Histone Deacetylase 4 (*HDAC4*). Consistent with previous studies (Adams et al., 1995; Tang et al., 2009), all of these markers were transcriptionally elevated in response to denervation surgery (Figures 1E,F). However, none of those markers was increased in either TA or GAST muscles of aged mice in the sham condition (Figures 1E,F), suggesting that age-related denervation did not occur significantly before the surgical denervation. In fact, we found that the induction of *Chrn* subunits, *Musk*, *Myog*, and *HDAC4* by denervation surgery was generally repressed in the aged muscles (Figures 1E,F). The potential implications of this observation will be discussed in the “Discussion” section.

### Effects of Aging on Protein Synthesis During Muscle Denervation

**Figure 2:**
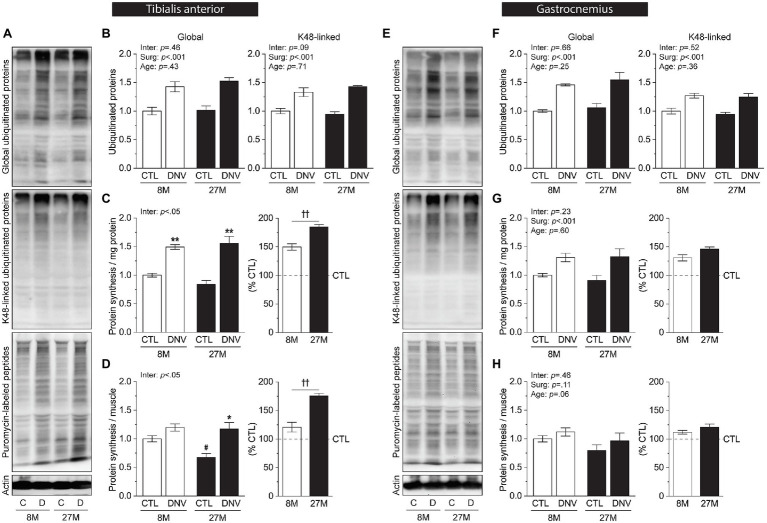
Effects of aging on protein synthesis during muscle denervation. Mice at 8months (8M) and 27months (27M) of age were subjected to denervation (right leg, DNV) and sham (contralateral left leg, CTL) surgeries, and TA and GAST muscles were collected 7days post-surgeries with puromycin injection. **(A)** Representative Western blots of TA samples. **(B–D)** Quantification of ubiquitinated proteins **(B)** and puromycin-labeled peptides per mg protein **(C)** or per muscle **(D)** in TA. **(E)** Representative Western blots of GAST samples. **(F–H)** Quantification of ubiquitinated proteins **(F)** and puromycin-labeled peptides per mg protein **(G)** or per muscle **(H)** in GAST. *n*=4–3 mice/group. Data are presented as mean±SEM. and expressed relative to 8M/CTL or as % of CTL (right panels in **C**,**D**,**G**,**H**). ^*^p<0.05 and ^**^p<0.01 vs. CTL by two-way mixed ANOVA. ^#^*p*<0.05 vs. 8M within the same surgery groups by two-way mixed ANOVA. ^††^*p*<0.01 by unpaired *t* test. Inter, interaction between surgery effects (Surg) and age effects by two-way mixed ANOVA.

The TA muscle-specific atrophy resistance in aged mice is interesting because its underlying mechanism could provide valuable information for developing therapies against denervation-induced muscle atrophy. To identify the potential mechanisms, we first asked if aging influenced protein degradation and protein synthesis in TA muscles during denervation. The levels of global and K48-linkage-specific polyubiquitination, which are readouts of ubiquitin-mediated protein degradation, were similarly increased in adult and aged mice in response to denervation (Figures 2A,B; Supplementary Figure S2A). On the other hand, the rate of protein synthesis as measured by SUnSET assay (Figure 2A) was elevated to a larger degree in aged mice than in adult mice when expressed per mg protein (Figure 2C) or elevated only in aged mice when expressed per muscle (Figure 2D). In GAST muscles, neither ubiquitin-mediated protein degradation nor protein synthesis rates were affected by aging during denervation (Figures 2E–H; Supplementary Figure S2B). These results indicate that aging promotes protein synthesis rates following denervation to confer resistance to denervation-induced atrophy in TA muscles.

### Effects of Aging on Ribosome Biogenesis During Muscle Denervation

**Figure 3:**
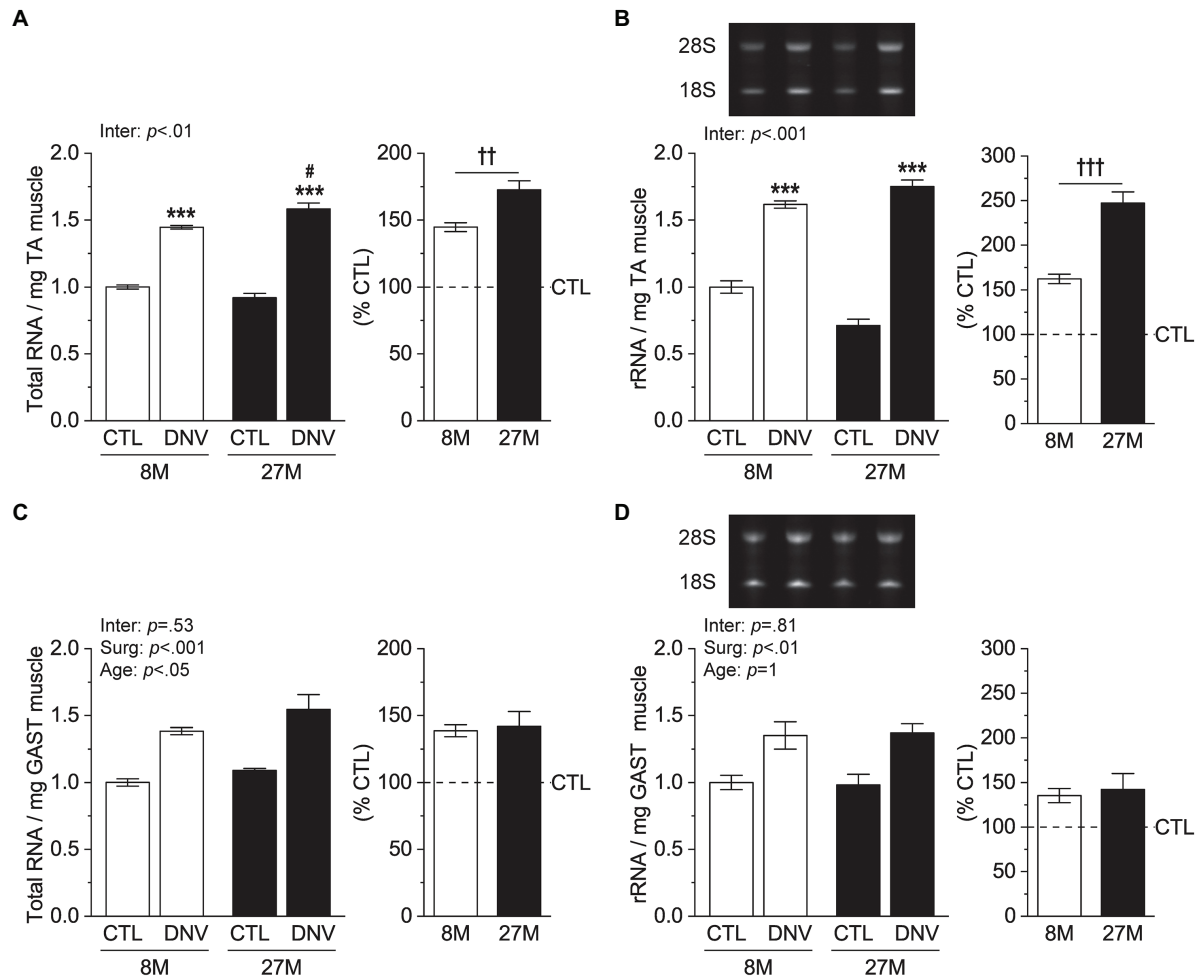
Effects of aging on ribosomal biogenesis during muscle denervation. Mice at 8months (8M) and 27months (27M) of age were subjected to denervation (right leg, DNV) and sham (contralateral left leg, CTL) surgeries, and TA and GAST muscles were collected 7days post-surgeries. **(A,B)** Quantification of total RNA **(A)** and 28S+18S rRNA **(B)** per mg muscle in TA. **(C,D)** Quantification of total RNA **(C)** and 28S+18S rRNA **(D)** per mg muscle in GAST. *n*=4–3 mice/group. Data are presented as mean±SEM. and expressed relative to 8M/CTL or as % of CTL (right panels). ^***^p<0.05 vs. CTL by two-way mixed ANOVA. ^#^*p*<0.05 vs. 8M within the same surgery groups by two-way mixed ANOVA. ^††^*p*<0.01 and ^†††^*p*<0.001 by unpaired *t* test. Inter, interaction between surgery effects (Surg) and age effects by two-way mixed ANOVA.

We next asked if the TA muscle-specific effect of aging on protein synthesis was associated with rRNA biogenesis, an essential process of making the protein synthesis machinery, ribosome. Total RNA content, which is composed of >85% of rRNA (O’Neil et al., 2013), was increased by denervation to a greater degree in aged mice than in adult mice (Figure 3A). Measurements of 28S+18S rRNA confirmed a greater increase in rRNA in the aged (Figure 3B). Consistent with the rate of protein synthesis, rRNA biogenesis in GAST muscles was not affected by aging during denervation (Figures 3C,D). Thus, these results strongly suggest that during denervation aging induces TA muscle-specific increase in protein synthesis by enhancing rRNA biogenesis.

### Effects of Aging on Akt Signaling During Muscle Denervation

**Figure 4:**
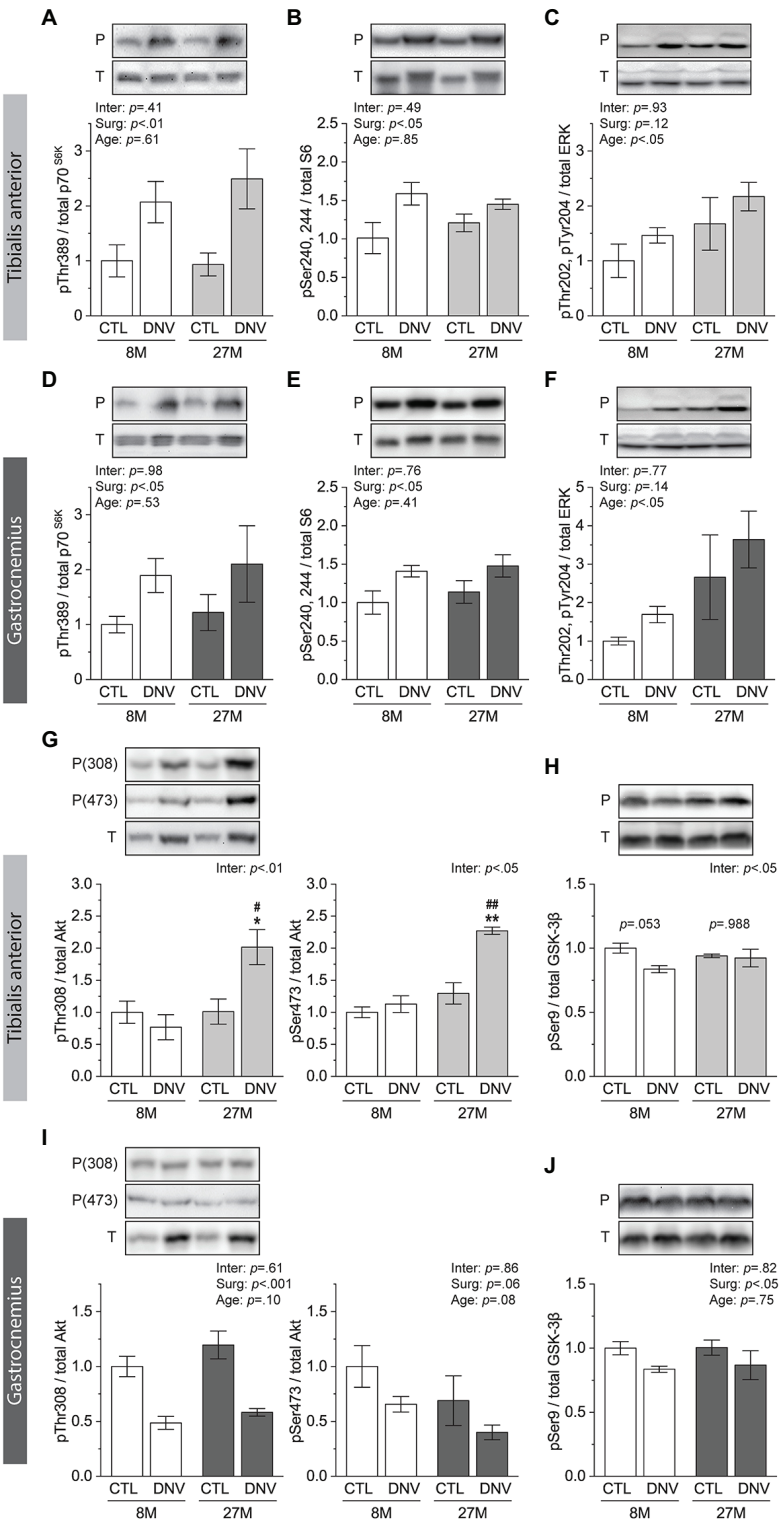
Effects of aging on mammalian target of rapamycin complex 1 (mTORC1), ERK, and Akt signaling during muscle denervation. Mice at 8months (8M) and 27months (27M) of age were subjected to denervation (right leg, DNV) and sham (contralateral left leg, CTL) surgeries, and TA and GAST muscles were collected 7days post-surgeries. **(A–C)** Quantification of Western blots for phosphorylated (P)/total (T) ratios of p70^S6K^ **(A)**, S6 **(B)**, and ERK **(C)** in TA. **(D–F)** Quantification of Western blots for phosphorylated (P)/total (T) ratios of p70^S6K^ **(D)**, S6 **(E)**, and ERK **(F)** in GAST. **(G,H)** Quantification of Western blots for phosphorylated (P)/total (T) ratios of Akt **(G)** and glycogen synthase kinase-3β (GSK-3β; **H**) in TA. **(I,J)** Quantification of Western blots for phosphorylated (P)/total (T) ratios of Akt **(I)** and GSK-3β **(J)** in GAST. *n*=5–3 mice/group. Data are presented as mean±SEM. and expressed relative to 8M/CTL. ^*^p<0.05 and ^**^p<0.01 vs. CTL by two-way mixed ANOVA. ^#^*p*<0.05 and ^##^*p*<0.01 vs. 8M within the same surgery groups by two-way mixed ANOVA. Inter, interaction between surgery effects (Surg) and age effects by two-way mixed ANOVA.

One of the key molecular regulators of rRNA biogenesis and protein synthesis is mTORC1 (Iadevaia et al., 2014). Signaling through mTORC1 has been reported to play an important role in the regulation of protein synthesis during denervation (Quy et al., 2013; You et al., 2021a). As such, we envisioned that mTORC1 may mediate the TA-specific effects of aging on rRNA biogenesis and protein synthesis during denervation. However, denervation-induced changes in mTORC1 signaling, revealed by the phosphorylation of the 70kDa ribosomal S6 protein kinase (p70^S6^K) and of the S6 protein, were not affected by aging in the TA (Figures 4A,B). ERK signaling, another important regulator of rRNA biogenesis and protein synthesis (Zhao et al., 2003; Stefanovsky and Moss, 2008), was also not affected by aging during denervation (Figure 4C). Similar observations were also made in GAST muscles (Figures 4D–F).

Signaling through Akt is known to promote rRNA biogenesis and protein synthesis independently of its role in activating mTORC1 (Proud, 2006; Chan et al., 2011; Nguyen and Mitchell, 2013), which raises the possibility that Akt may be involved in the age- and TA-specific changes in the anabolic events during denervation. Indeed, in the TA, phosphorylation of Akt on Thr308 and Ser473, both indicating Akt activation, were increased only in the aged muscles during denervation (Figure 4G). Consistently, aging nearly significantly prevented the denervation-induced decrease in the phosphorylation of GSK-3β on Ser9, a direct substrate of Akt (Figure 4H). These age-dependent changes in Akt and GSK-3β phosphorylation did not occur in GAST muscles (Figures 4I,J). Combined, these results suggest that during denervation aging promotes rRNA biogenesis and protein synthesis specifically in the TA muscle through Akt signaling.

## Discussion

Although efforts have been actively made in understanding age-related motoneuron loss that accelerates sarcopenia, little is known about whether aged/sarcopenic muscles *per se* respond differently to denervation than younger muscles. In this study, we found that aged muscle with clear signs of sarcopenia is not more susceptible to denervation atrophy than younger muscle and rather shows unexpected atrophy resistance in a specific type of muscle. This unique muscle-type-specific atrophy resistance was tightly associated with a cascade of anabolic events involving enhanced Akt signaling, rRNA biogenesis, and protein synthesis during denervation. Our results expand the current understanding of age-dependent stress responses and provide insights that can help develop better strategies to combat sarcopenia.

It has been reported that aged muscles at 24-month-old have only a very modest level of complete denervation (Liu et al., 2017). Consistently, we did not observe any marked increase in markers of denervation, such as *Chrn* subunits and *Musk*, in muscles of 27-month-old mice. Upon surgical denervation, those post-synaptic genes were elevated in aged muscles, but interestingly, the magnitude of the increase was overall less than in adult muscles. Denervation activates *Chrn* genes likely to compensate for the loss of functional CHRNs (Bevan and Steinbach, 1983; Adams et al., 1995; Guyon et al., 1998), and maintaining active CHRN signaling in denervated muscle is known to both prevent atrophy and promote re-innervation by nearby neurons (Cisterna et al., 2020). The reduced ability of the aged muscle to induce a compensatory expression of *Chrns* and other post-synaptic genes upon denervation may contribute to the accelerated atrophy and failed re-innervation when complete denervation of motor units occurs in advanced age (Aare et al., 2016).

Signaling through Akt has long been recognized as an inhibitor of muscle atrophy during denervation (Bodine et al., 2001; Castets et al., 2019). However, whether and how Akt may regulate denervation atrophy in aged muscle has been unknown. We show that, during denervation, Akt signaling is enhanced with aging in a specific type of muscle (i.e., TA) that exhibits atrophy resistance. This selective increase in Akt signaling was not accompanied by an increase in mTORC1 signaling, which is consistent with a recent study showing no changes in S6 phosphorylation in constitutively active Akt1 transgenic mice during denervation (Castets et al., 2019). Instead, the increase in Akt signaling was followed by the prevention of the denervation-induced decrease in the phosphorylation of GSK-3β. Phosphorylation-dependent inactivation of GSK-3β inhibits phosphorylation of the key translation initiation factor eIF2B, contributing to eIF2B activation in muscles (Jefferson et al., 1999; Sutherland, 2011). Thus, prevention of the denervation-induced decrease in GSK-3β phosphorylation can promote protein synthesis during denervation. Akt can also promote protein synthesis independently of mTORC1 through rRNA biogenesis (Chan et al., 2011; Nguyen and Mitchell, 2013). Indeed, we found that the increase in Akt signaling in aged TA muscles coincided with an age-dependent and TA-specific increase in the levels of 28S and 18S rRNAs during denervation. Therefore, our results suggest that an increase in Akt signaling may alleviate denervation-induced atrophy of aged muscles *via* mTORC1-independent increases in both translational efficiency and translational capacity.

An interesting but unanswered question in this study is how the atrophy resistance and Akt activation occurred only in the aged TA muscle. Different fiber type compositions in aged muscles might be involved, but the observations that two similar fast muscle types, TA and GAST, showed different phenotypes, and two opposite muscle types, EDL (fast) and SOL (slow), showed similar phenotypes, do not seem to support this possibility. For more accurate probing of this possibility, fiber typing analysis will be required. As an alternative mechanism, we examined some of the key components of IGF-1 signaling upstream of Akt in TA muscles. These included the levels of IGF-1 receptor autophosphorylation (activation), insulin receptor substrate-1 tyrosine phosphorylation, phosphatidylinositol 3-kinase (PI3K) p85 subunit, and PTEN (Luo et al., 2005; Schiaffino and Mammucari, 2011). Denervation induced changes in those factors, but there was no aging effect on the alterations (Supplementary Figure S3). Other regulatory mechanisms of Akt, such as phosphatases or other types of post-translational modifications (Chan et al., 2014), could be influenced by aging during denervation in the TA. Identifying the mechanisms of the unique atrophy resistance and Akt activation in the aged muscle with denervation will help develop future therapies targeting sarcopenia.

## Data Availability Statement

The raw data supporting the conclusions of this article will be made available by the authors, without undue reservation.

## Ethics Statement

The animal study was reviewed and approved by the Institutional Animal Care and Use Committee at the University of Illinois at Urbana-Champaign.

## Author Contributions

J-SY designed research, performed experiments, analyzed data, and drafted the manuscript. JC acquired funding and edited the manuscript. All authors contributed to the article and approved the submitted version.

## Funding

This work was supported by a grant from the National Institutes of Health to JC (R01AR048914).

## Conflict of Interest

The authors declare that the research was conducted in the absence of any commercial or financial relationships that could be construed as a potential conflict of interest.

## Publisher’s Note

All claims expressed in this article are solely those of the authors and do not necessarily represent those of their affiliated organizations, or those of the publisher, the editors and the reviewers. Any product that may be evaluated in this article, or claim that may be made by its manufacturer, is not guaranteed or endorsed by the publisher.
