# Supplementary material for: Aging Does Not Exacerbate Muscle Loss During Denervation and Lends Unique Muscle-Specific Atrophy Resistance With Akt Activation
Source: Front Physiol. 2021 Nov 30;12:779547. doi: 10.3389/fphys.2021.779547 (PMC8669767; doi:10.3389/fphys.2021.779547)
Supplement: Supplementary file 1 [file Data_Sheet_1.PDF]

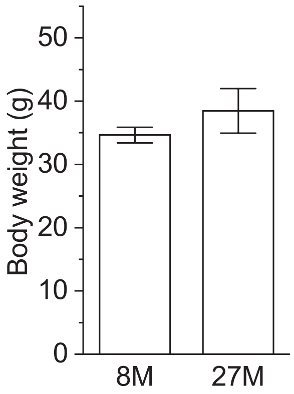

Supplementary Figure 1. Mouse body weights. Related to Figure 1.

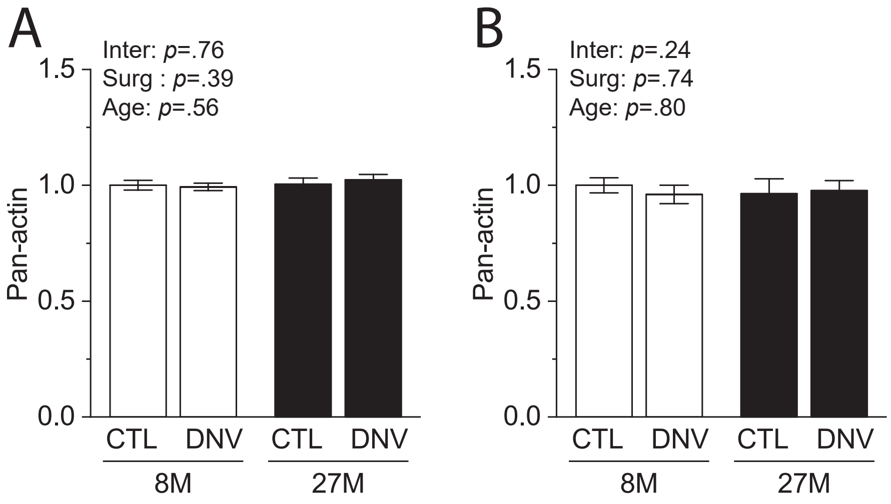

Supplementary Figure 2. Quantification of pan-actin. Related to Figure 2. (A) Tibialis anterior muscles. (B) Gastrocnemius muscles. n = 4-3 mice/group. Data are presented as mean ± SEM. and expressed relative to 8M/CTL. Inter, interaction between surgery effects (Surg) and age effects by two-way mixed ANOVA.

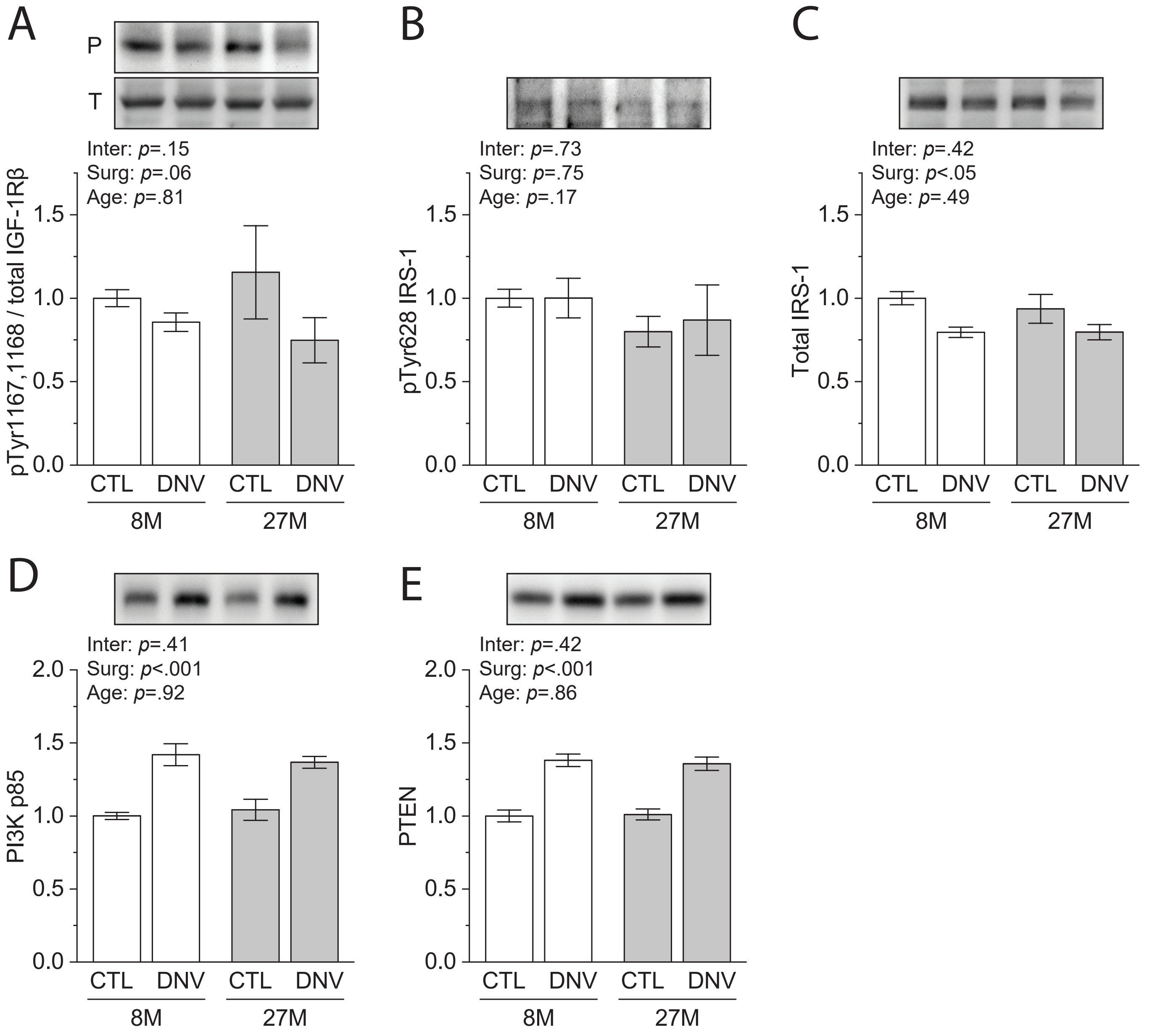

Supplementary Figure 3. Effects of aging on proximal IGF-1 signaling during denervation. Mice at 8 months (8M) and 27 months (27M) of age were subjected to denervation (right leg, DNV) and sham (contralateral left leg, CTL) surgeries, and tibialis anteria muscles were collected 7 days post-surgeries. (A-E) Quantification of Western blots for phosphorylated (P)/total (T) ratios of IGF-1R β (A) and the levels of phosphorylated IRS-1 (B), total IRS-1 (C), the p85 subunit of PI3K (D), and PTEN (E). n = 4-3 mice/group. Data are presented as mean ± SEM. and expressed relative to 8M/CTL. Inter, interaction between surgery effects (Surg) and age effects by two-way mixed ANOVA.
